# Publisher Correction: Diattenuation Imaging reveals different brain tissue properties

**DOI:** 10.1038/s41598-019-42189-8

**Published:** 2019-04-19

**Authors:** Miriam Menzel, Markus Axer, Katrin Amunts, Hans De Raedt, Kristel Michielsen

**Affiliations:** 1 Institute of Neuroscience and Medicine (INM-1), Forschungszentrum Jülich GmbH, 52425 Jülich, Germany; 2 Department of Physics, RWTH Aachen University, 52056 Aachen, Germany; 3 Cécile and Oskar Vogt Institute for Brain Research, University Hospital Düsseldorf, University of Düsseldorf, 40204 Düsseldorf, Germany; 4 Zernike Institute for Advanced Materials, University of Groningen, 9747AG Groningen, The Netherlands; 5 Jülich Supercomputing Centre, Forschungszentrum Jülich GmbH, 52425 Jülich, Germany

Correction to: *Scientific Reports* 10.1038/s41598-019-38506-w, published online 13 February 2019

This Article contains typographical errors introduced during the publication process.

In the Results section, under subheading ‘Simulation Studies’,

“The magnitude of *D*_S_ is related to the strength of the diattenuation ($|{\mathscr{D}}_{{\rm{S}}}|\approx |\mathscr{D}|$), the sign indicates the phase *φ*_D_ (cf. equation (1)): Positive values (*D*_S_ > 0 $\iff$ *I*_x_ > *I*_y_) correspond to regions with *D*^+^ and are shown in green (the transmitted light intensity becomes maximal when the light is polarised parallel to the fibre structure, i.e. in the x-direction)”.

should read:

“The magnitude of *D*_S_ is related to the strength of the diattenuation (|*D*_S_| ≈ $|\mathscr{D}|$), the sign indicates the phase *φ*_D_ (cf. equation (1)): Positive values (*D*_S_ > 0 $\iff$ *I*_x_ > *I*_y_) correspond to regions with *D*^+^ and are shown in green (the transmitted light intensity becomes maximal when the light is polarised parallel to the fibre structure, i.e. in the x-direction)”.

In the methods section, under subheading ‘Preparation of brain sections’,

“The frozen brains were cut with a cryostat microtome (*Leica Microsystems*, Germany) into sections of 60 m”.

should read:

“The frozen brains were cut with a cryostat microtome (*Leica Microsystems*, Germany) into sections of 60 µm”.

In the same section, under subheading ‘Measurements with prototypic polarising microscope’,

“The microscope objective has a 4× magnification and a numerical aperture of 0.2, yielding a pixel size in object space of about 1.8 m”.

should read:

“The microscope objective has a 4× magnification and a numerical aperture of 0.2, yielding a pixel size in object space of about 1.8 µm”.

In the same section, under subheading ‘FDTD simulations’,

“The propagation of the polarised light wave through the sample was computed by TDME3DTM,–a massively parallel three-dimensional Maxwell Solver^54–56^ based on a finite-difference time-domain (FDTD) algorithm^36,57^”.

should read:

“The propagation of the polarised light wave through the sample was computed by *TDME3D*^TM^, –a massively parallel three-dimensional Maxwell Solver^54–56^ based on a finite-difference time-domain (FDTD) algorithm^36,57^”.

and,

“The sample contains the fibre configuration (30 × 30 × 30 μm^3^, see above) and 0.5 m thick layers of glycerine solution on top and at the bottom”.

should read:

“The sample contains the fibre configuration (30 × 30 × 30 μm^3^, see above) and 0.5 µm thick layers of glycerine solution on top and at the bottom”.

and,

“The propagation of the light wave through the sample (fibre configuration) was computed by TDME3D, yielding a superposition of monochromatic plane waves with different wave vectors”.

should read:

“The propagation of the light wave through the sample (fibre configuration) was computed by TDME3D^TM^, yielding a superposition of monochromatic plane waves with different wave vectors”.

In the same section, under subheading ‘Code availability’,

“For the FDTD simulations, we used TDME3D, a massively parallel Maxwell solver^54–56^. The software is property of EMBD (European Marketing and Business Development BVBA)”.

should read:

“For the FDTD simulations, we used TDME3D^TM^, a massively parallel Maxwell solver^54–56^. The software is property of EMBD (European Marketing and Business Development BVBA)”.

Finally, in the Acknowledgements section,

“This project has received funding from the Helmholtz Association portfolio theme ‘*Supercomputing and Modeling for the Human Brain*’, from the European Union’s Horizon 2020 Research and Innovation Programme under Grant Agreement No. 720270 (HBP SGA1) and 785907 (HBP SGA2), and from the National Institutes of Health under grant agreements No. R01MH092311 and 5P40OD010965”.

should read:

“This project has received funding from the Helmholtz Association portfolio theme ‘*Supercomputing and Modeling for the Human Brain*’, from the European Union’s Horizon 2020 Research and Innovation Programme under Grant Agreement No. 7202070 (HBP SGA1) and 785907 (HBP SGA2), and from the National Institutes of Health under grant agreements No. R01MH092311 and 5P40OD010965”.

