# Supplementary material for: Diattenuation Imaging reveals different brain tissue properties
Source: Sci Rep. 2019 Feb 13;9:1939. doi: 10.1038/s41598-019-38506-w (PMC6374401; doi:10.1038/s41598-019-38506-w)
Supplement: Supplementary file 1 — Supplementary Information [file 41598_2019_38506_MOESM1_ESM.pdf]

Diattenuation Imaging reveals different brain tissue properties

Miriam Menzel[1,2,*], Markus Axer[1], Katrin Amunts[1,3],
Hans De Raedt[4], Kristel Michielsen[5,2]

1. Institute of Neuroscience and Medicine (INM-1), Forschungszentrum Jülich GmbH, 52425 Jülich, Germany
2. Department of Physics, RWTH Aachen University, 52056 Aachen, Germany
3. Cécile and Oskar Vogt Institute for Brain Research, University Hospital Düsseldorf, University of Düsseldorf, 40204 Düsseldorf, Germany
4. Zernike Institute for Advanced Materials, University of Groningen, 9747AG Groningen, the Netherlands
5. Jülich Supercomputing Centre, Forschungszentrum Jülich GmbH, 52425 Jülich, Germany

∗ corresponding author email: m.menzel@fz-juelich.de

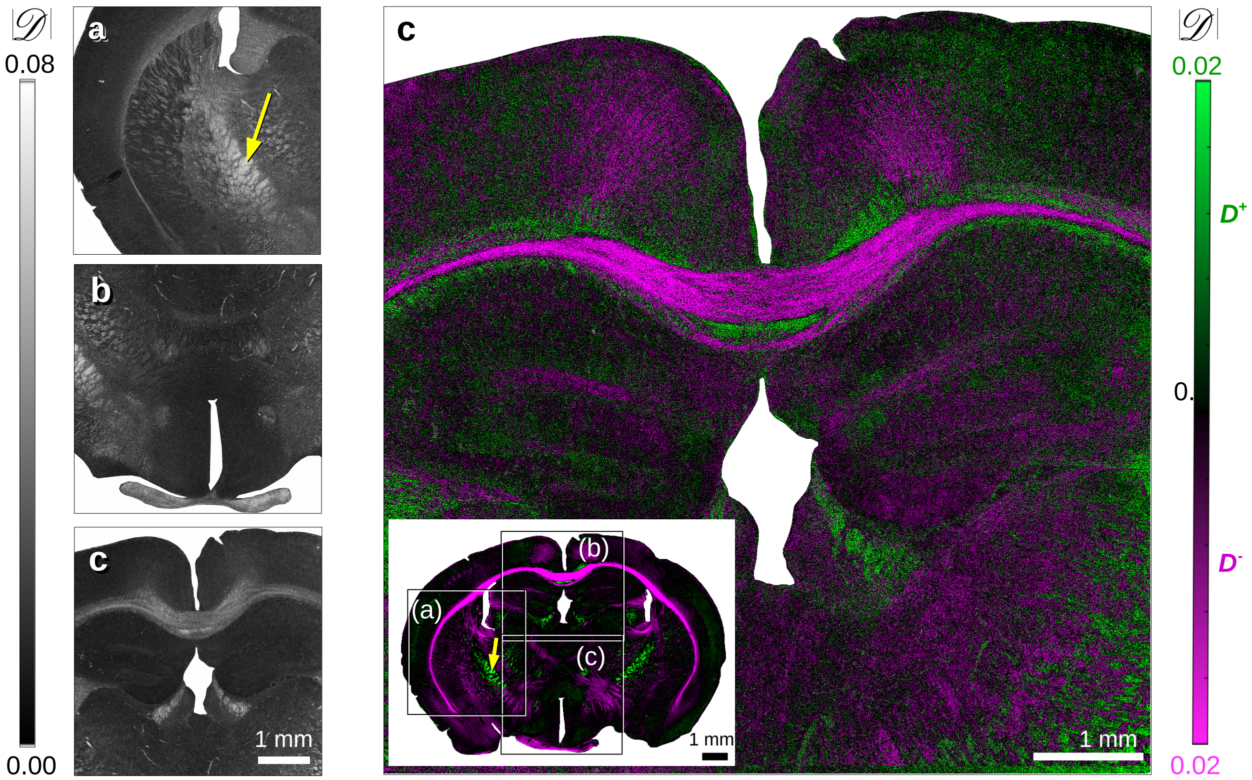

Supplementary Figure 1. High-resolution diattenuation measurement. The figure shows the diattenuation images of a coronal mouse brain section (cf. Fig. 2a) obtained from polarimetric measurements performed with the LAP (whole brain section, inset) and with a prototypic polarising microscope realised on an optical bench[1] (enlarged areas a–c), see Methods. The measurement with the LAP was performed with an effective object-space resolution of 14 µm / px one day after tissue embedding. The measurements with the polarising microscope were performed with a pixel size of about 1.8 µm two days after tissue embedding. The gray-scale images show the strength of the diattenuation signal $|\mathscr{D}|$, the coloured images show the diattenuation values belonging to regions with diattenuation of type D^+ ($\varphi_\mathrm{D} - \varphi_\mathrm{P} \in [-20°, 20°]$, in green) and regions with diattenuation of type D^- ($\varphi_\mathrm{D} - \varphi_\mathrm{P} \in [70°, 110°]$, in magenta). The yellow arrows mark the maximum diattenuation measured with the microscope. The diattenuation in a region of 10×10 pixels ($|\mathscr{D}| \approx 9.9\,\%$) is much larger than the diattenuation of the same region measured with the LAP ($|\mathscr{D}| \approx 3.6\,\%$). However, large diattenuation values and diattenuation of type D^+ or D^- are observed in similar regions, i.e. the diattenuation effects do not depend on the optical resolution of the imaging system.

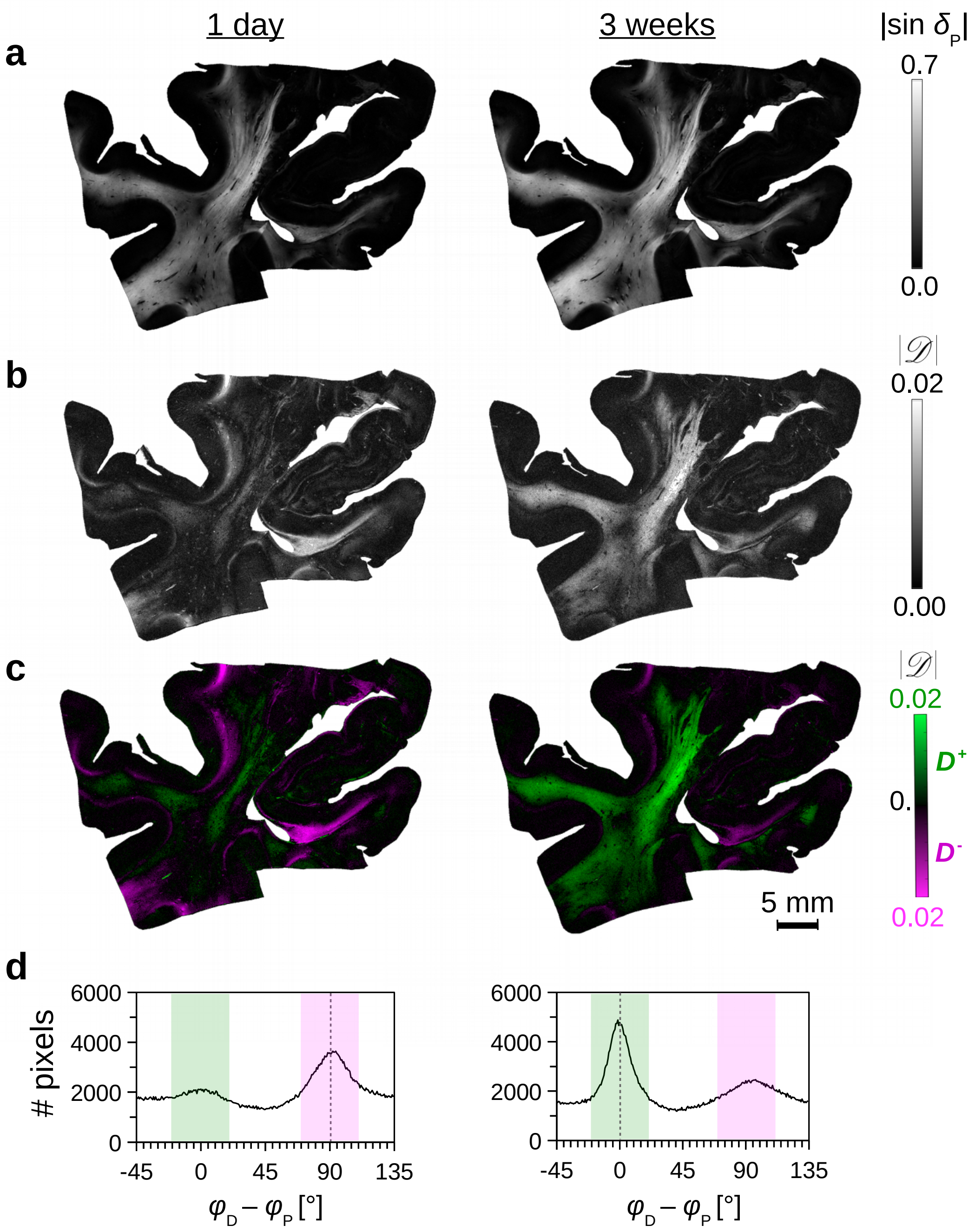

Supplementary Figure 2. Dependence of diattenuation on embedding time for human brain tissue (coronal, 60 µm thick section of a human temporal lobe measured one day and three weeks after embedding the brain section). **a** Strength of the measured birefringence signal $|\sin\delta_{\mathrm{P}}|$. **b** Strength of the measured diattenuation signal $|\mathscr{D}|$. **c–d** Diattenuation images and $(\varphi_{\mathrm{D}} - \varphi_{\mathrm{P}})$ histograms. Diattenuation values that belong to regions with diattenuation of type D^{+} $(\varphi_{\mathrm{D}} - \varphi_{\mathrm{P}} \in [-20^{\circ}, 20^{\circ}])$ are shown in green, diattenuation values that belong to regions with diattenuation of type D^{-} $(\varphi_{\mathrm{D}} - \varphi_{\mathrm{P}} \in [70^{\circ}, 110^{\circ}])$ are shown in magenta. The values $\{|\mathscr{D}|, \varphi_{\mathrm{D}}, \varphi_{\mathrm{P}}\}$ were determined from polarimetric measurements with the LAP (see Methods) with an effective object-space resolution of 40 µm / px. After three weeks, much more regions show diattenuation of type D^{+} (green) and the main peak of $(\varphi_{\mathrm{D}} - \varphi_{\mathrm{P}})$ is shifted from about 90° to 0°.

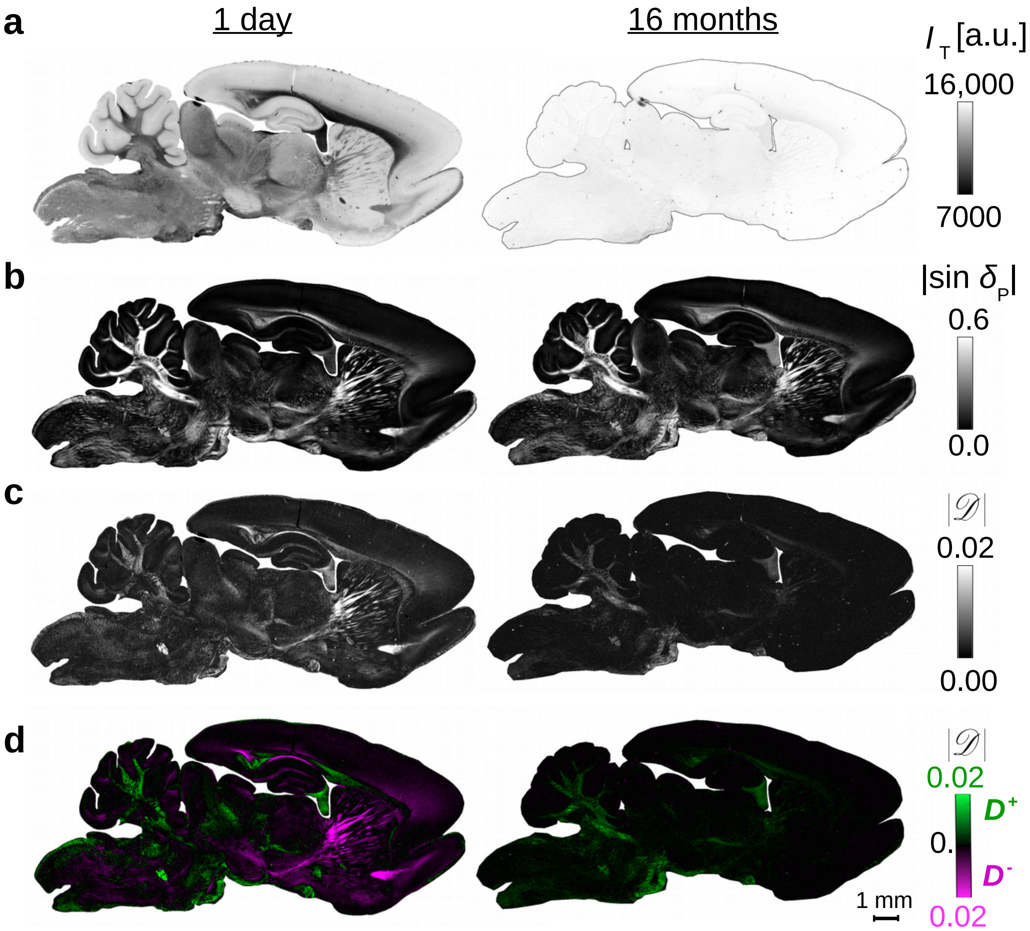

Supplementary Figure 3. Dependence of transmittance, birefringence, and diattenuation on embedding time (sagittal rat brain section in Figs. 1c and 2a measured one day and 16 months after embedding). **a** Average transmitted light intensity (transmittance I_T). **b** Strength of the measured birefringence signal $|\sin\delta_P|$. **c** Strength of the measured diattenuation signal $|\mathscr{D}|$. **d** Diattenuation images: $|\mathscr{D}|$ values belonging to regions with diattenuation of type D^+ ($\varphi_D - \varphi_P \in [-20°, 20°]$) are shown in green, values belonging to regions with diattenuation of type D^- ($\varphi_D - \varphi_P \in [70°, 110°]$) are shown in magenta). The values $\{I_T, |\sin\delta_P|, |\mathscr{D}|, \varphi_D, \varphi_P\}$ were determined from polarimetric measurements with the LAP (see Methods) with an effective object-space resolution of 14 µm / px. After 16 months, the transmittance fades out and regions with D^- vanish, while the birefringence barely changes.

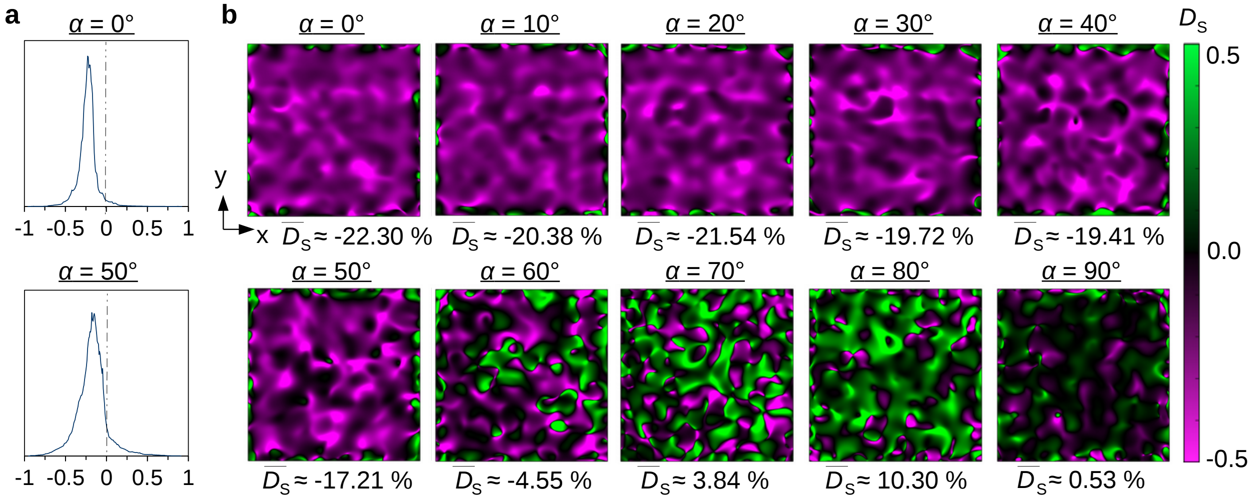

Supplementary Figure 4. Simulated diattenuation images for different fibre inclination angles (for the bundle of densely grown fibres, see Fig. 4a). **a** Histograms of D_S for inclination $\alpha = 0^\circ$ (mean: $-22\,\% \pm 12\,\%$) and $\alpha = 50^\circ$ (mean: $-17\,\% \pm 19\,\%$). **b** Diattenuation images D_S and mean values $\overline{D_S}$ for all inclination angles. Positive diattenuation values (representing type D^+) are displayed in green, negative diattenuation values (representing type D^-) are displayed in magenta. The diattenuation images were obtained from simulated diattenuation measurements with light polarised along the x-axis (I_x) and along the y-axis (I_y): $D_S = (I_x - I_y)/(I_x + I_y)$ (see Methods). The diattenuation is mostly negative (magenta) for fibres with small inclination angles ($\alpha \leq 50^\circ$) and becomes more positive (green) for steeper fibres ($\alpha > 60^\circ$).

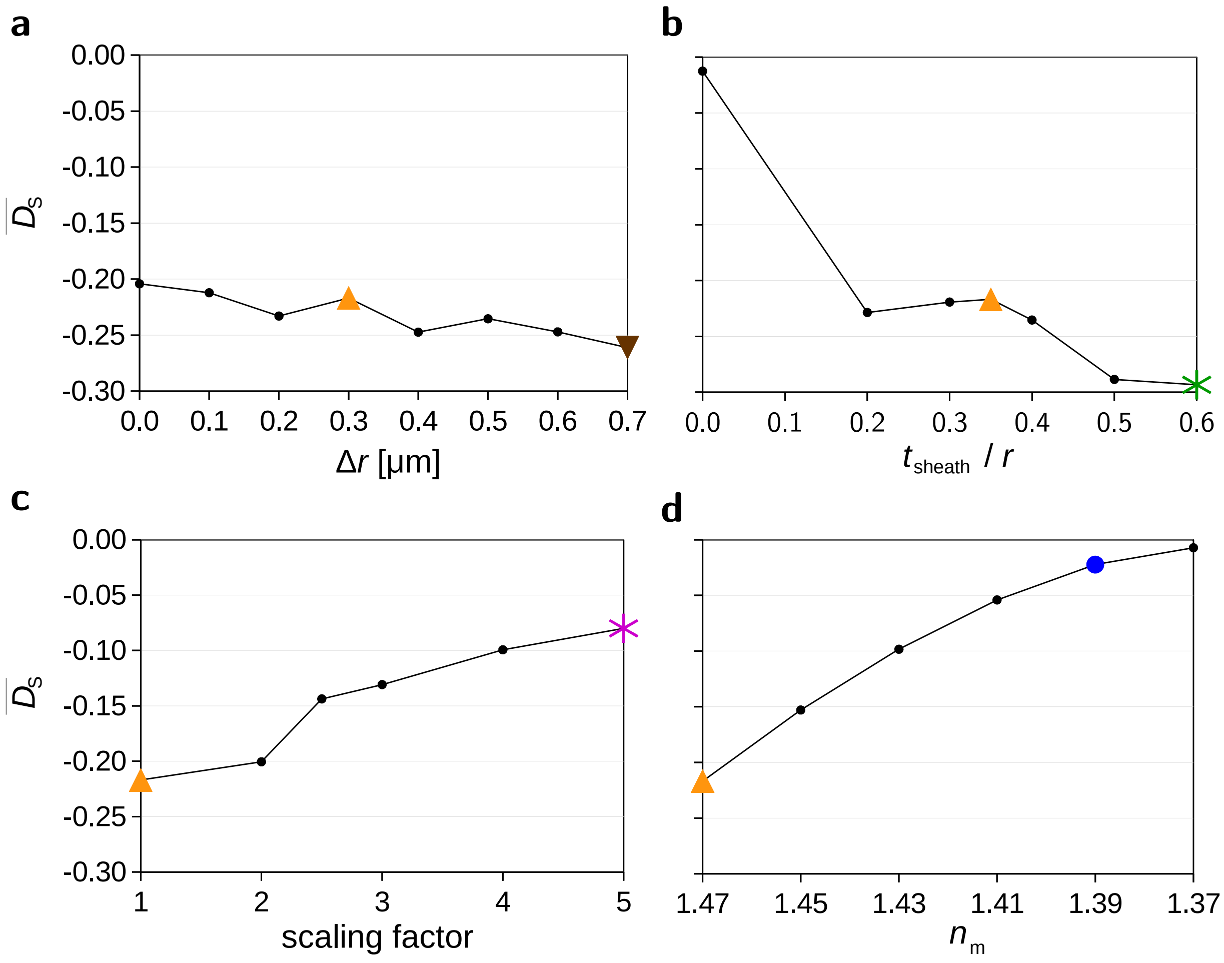

Supplementary Figure 5. Simulated diattenuation values for different fibre properties. The figure shows the mean diattenuation values $\overline{D_S}$ obtained from simulations of the horizontal bundle of densely grown fibres (cf. Fig. 4a with $\alpha = 0°$) for different fibre properties (see Methods): **a** different fibre radius distributions $r \in [r_{min}, r_{max}]$ with $\Delta r \equiv r_{max} - r_{min}$ and $(r_{max} + r_{min})/2 = 0.65\,\mu m$, **b** different myelin sheath thicknesses t_{sheath} (relative to the fibre radius r), **c** different scaling factors (fibre sizes), **d** different myelin refractive indices n_m. The values belonging to the graphs in Fig. 4c are highlighted in the respective colours: bundle of densely grown fibres with $\{r \in [0.5, 0.8]\,\mu m$, $t_{sheath} = 0.35\,r$, $n_m = 1.47\}$ (orange triangle), bundle with broad distribution of radii $r \in [0.3, 1.0]\,\mu m$ (brown triangle), bundle with thick myelin sheaths $t_{sheath} = 0.6\,r$ (green star), bundle with large fibres $r \in [2.5, 4.0]\,\mu m$ (magenta star), bundle with reduced myelin refractive index $n_m = 1.39$ (blue circle). The mean diattenuation $\overline{D_S}$ becomes less negative with smaller fibre radius distribution, smaller myelin sheath thickness, larger fibre size (scaling factor), and smaller myelin refractive index.

Supplementary Note. Analytical Model of Dichroism

Dichroism in Uniaxial Absorbing Materials

In uniaxial absorbing materials with high symmetry, the birefringence (anisotropic refraction) and the dichroism (anisotropic absorption) can be described by a complex retardance with shared principal axes.

Defining a complex refractive index

$$n' \equiv n + \mathrm{i}\,\kappa \equiv n(1 + \mathrm{i}\,\hat{\kappa}) \tag{1}$$

and assuming weak absorption ($\kappa \ll n$), the absorption of the extraordinary light wave ($\hat{\kappa}_\mathrm{e}$) is given by (cf. BORN & WOLF,[2] Sec. 15.6.1):

$$v_\mathrm{e}^2\,\hat{\kappa}_\mathrm{e} = v_\mathrm{o}^2\,\hat{\kappa}_\mathrm{o}\cos^2\theta + v_\mathrm{E}^2\,\hat{\kappa}_\mathrm{E}\sin^2\theta, \tag{2}$$

where θ is the angle between the optic axis (symmetry axis) and the wave vector, v is the phase velocity of the light, and the indices "o", "e", and "E" denote the properties of the ordinary wave (polarised perpendicularly to the optic axis), the extraordinary wave, and the principal extraordinary wave (polarised parallel to the optic axis), respectively.

Writing $\hat{\kappa} = \kappa/n$, $v = c/n$ (c is the velocity of light in vacuum), and defining $\Delta n \equiv n_\mathrm{E} - n_\mathrm{o}$, $\Delta n_\mathrm{e} \equiv n_\mathrm{e} - n_\mathrm{o}$, $\Delta\kappa \equiv \kappa_\mathrm{E} - \kappa_\mathrm{o}$, equation (2) yields:

$$\kappa_\mathrm{e} = \frac{n_\mathrm{e}^3}{n_\mathrm{o}^3}\,\kappa_\mathrm{o}\cos^2\theta + \frac{n_\mathrm{e}^3}{n_\mathrm{E}^3}\,\kappa_\mathrm{E}\sin^2\theta \tag{3}$$

$$= \underbrace{\frac{(n_\mathrm{o}+\Delta n_\mathrm{e})^3}{n_\mathrm{o}^3}}_{f_1(\Delta n_\mathrm{e})}\,\kappa_\mathrm{o}\cos^2\theta + \underbrace{\frac{(n_\mathrm{o}+\Delta n_\mathrm{e})^3}{(n_\mathrm{o}+\Delta n)^3}}_{f_2(\Delta n_\mathrm{e},\Delta n)}\,(\kappa_\mathrm{o}+\Delta\kappa)\sin^2\theta. \tag{4}$$

The birefringence of biological tissue[3] ($|\Delta n| = 0.001$–0.01) is small compared to its refractive index values[4] ($n = 1.3$–1.5). In this case, a first order Taylor expansion (in 2D) can be applied to the functions f_1 and f_2 in $\{\Delta n_\mathrm{e} = 0,\ \Delta n = 0\}$:

$$f_1(\Delta n_\mathrm{e}) = f(0) + f'(0)\,\Delta n_\mathrm{e} + \dots \tag{5}$$

$$= 1 + \frac{3}{n_\mathrm{o}}\,\Delta n_\mathrm{e} + \dots \tag{6}$$

$$f_2(\Delta n_\mathrm{e},\,\Delta n) = f(0,0) + \frac{\partial}{\partial\Delta n_\mathrm{e}} f(0,0)\,\Delta n_\mathrm{e} + \frac{\partial}{\partial\Delta n} f(0,0)\,\Delta n + \dots \tag{7}$$

$$= 1 + \frac{3}{n_\mathrm{o}}\,(\Delta n_\mathrm{e} - \Delta n) + \dots \tag{8}$$

For $|\Delta n| \ll n$, the following approximation can be used, see MENZEL *et al.*,[5] equation A5:

$$\Delta n_{\mathrm{e}} \approx \Delta n \sin^2\theta. \tag{9}$$

Inserting equations (6) and (8) into equation (4) and using equation (9) yields:

$$\kappa_{\mathrm{e}} \approx \left(1 + \frac{3}{n_{\mathrm{o}}}\Delta n \sin^2\theta\right)\kappa_{\mathrm{o}}\cos^2\theta + \left(1 - \frac{3}{n_{\mathrm{o}}}\Delta n \cos^2\theta\right)(\kappa_{\mathrm{o}} + \Delta\kappa)\sin^2\theta \tag{10}$$

$$= \kappa_{\mathrm{o}} + \left(1 - \frac{3}{n_{\mathrm{o}}}\Delta n \cos^2\theta\right)\Delta\kappa \sin^2\theta. \tag{11}$$

For $|\Delta n| \leq 0.01$ and $n \geq 1.3$, the term in round brackets in equation (11) is > 0.97 and $\kappa_{\mathrm{e}} \approx \kappa_{\mathrm{o}} + \Delta\kappa \sin^2\theta$.

Thus, in uniaxial absorbing materials with small birefringence ($|\Delta n| \ll n$) and weak absorption ($\kappa \ll n$) like brain tissue, the dichroism (anisotropic absorption) depends on the angle θ between the wave vector and the optic axis, just like the birefringence in equation (9):

$$\Delta\kappa_{\mathrm{e}} \equiv \kappa_{\mathrm{e}} - \kappa_{\mathrm{o}} \approx \Delta\kappa \sin^2\theta\,, \quad \Delta\kappa \equiv \kappa_{\mathrm{E}} - \kappa_{\mathrm{o}}. \tag{12}$$

Inclination Dependence of Dichroism

The diattenuation of a brain section is computed via:

$$D = \frac{I_{\parallel} - I_{\perp}}{I_{\parallel} + I_{\perp}}, \tag{13}$$

with $I_{\parallel}$ and $I_{\perp}$ being the (maximum and minimum) transmitted light intensities for light polarised parallel and perpendicularly to the projection of the optic axis (predominant orientation of the nerve fibres) onto the section plane.

For light propagating through a brain section of thickness d, the transmitted light intensity is given by the Beer-Lambert law:

$$I = I_0\,\mathrm{e}^{-\mu d}, \tag{14}$$

where I_0 is the ingoing light intensity and μ the attenuation coefficient of the material which

depends on the direction of polarisation. With this definition, equation (13) can be written as:

$$D = \frac{e^{-d\mu_\parallel} - e^{-d\mu_\perp}}{e^{-d\mu_\parallel} + e^{-d\mu_\perp}} = \frac{e^{d(\mu_\perp-\mu_\parallel)/2} - e^{-d(\mu_\perp-\mu_\parallel)/2}}{e^{d(\mu_\perp-\mu_\parallel)/2} + e^{-d(\mu_\perp-\mu_\parallel)/2}} \tag{15}$$

$$= \tanh\left(d\,\frac{\mu_\perp - \mu_\parallel}{2}\right). \tag{16}$$

To estimate how the dichroism of brain tissue depends on the nerve fibre inclination, we assume that the diattenuation is solely caused by anisotropic absorption, neglecting any scattering. In this case, the attenuation coefficient is given by the absorption coefficient (DEMTRÖDER,[6] p. 227):

$$\mu = K \equiv \frac{4\pi\kappa}{\lambda}, \tag{17}$$

where κ is the imaginary part of the refractive index in the medium (see equation (1)) and λ the wavelength of the light. In a similar way, we define the absorption coefficient of the ordinary wave ($K_\perp \equiv 4\pi\kappa_o/\lambda$) and of the extraordinary wave ($K_\parallel \equiv 4\pi\kappa_e/\lambda$), taking into account that the ordinary wave is polarised perpendicularly to the optic axis while the extraordinary wave is polarised parallel to the projection of the optic axis onto the section plane.

With these definitions, the diattenuation caused by anisotropic absorption (dichroism) can be written as:

$$D_K \overset{(16)}{=} \tanh\left(\frac{d}{2}\left(K_\perp - K_\parallel\right)\right) \overset{(17)}{=} \tanh\left(\frac{2\pi d}{\lambda}\left(\kappa_o - \kappa_e\right)\right) \overset{(12)}{\approx} \tanh\left(-\frac{2\pi d}{\lambda}\Delta\kappa \sin^2\theta\right), \tag{18}$$

where θ is the angle between the optic axis (nerve fibre orientation) and the wave vector (direction of propagation).

Assuming that the brain section is illuminated under normal incidence, the out-of-plane inclination angle of the fibres is given by: $\alpha = 90° - \theta$. Thus, the dichroism of brain tissue decreases with increasing fibre inclination angle and does not change its sign (cf. Fig. 4c):

$$D_K \approx \tanh\left(-\frac{2\pi d}{\lambda}\Delta\kappa \cos^2\alpha\right). \tag{19}$$

Freshly embedded brain sections show diattenuation of both types D^+ and D^- (see Fig. 2). This suggests that the diattenuation is not only caused by dichroism (anisotropic absorption) but also by anisotropic scattering of light. With increasing time after tissue embedding, the brain sections become transparent (see Supplementary Fig. 3a), i. e. the scattering is expected to decrease due to an equalisation of the refractive indices. Thus, the diattenuation of type D^+ that

is observed in brain sections with long embedding time (see Fig. 3 and Supplementary Fig. 2) is presumably caused by dichroism, i.e. $D_K > 0 \Leftrightarrow I_{\parallel} > I_{\perp}$. This means that the absorption becomes maximal (the transmitted light intensity becomes minimal) when the light is polarised perpendicularly to the fibre axis, i.e. in the plane of the radially oriented lipid molecules in the myelin sheaths. This suggests that dichroism of brain tissue is mainly caused by the myelin lipids and therefore not expected to change with increasing time after tissue embedding, just like the birefringence (see Supplementary Fig. 3b).

Supplementary References

1. Wiese, H. *Enhancing the Signal Interpretation and Microscopical Hardware Concept of 3D Polarized Light Imaging*. Ph.D. thesis, University of Wuppertal (2016).

2. Born, M. & Wolf, E. *Principles of Optics – Electromagnetic Theory of Propagation, Interference and Diffraction of Light* (Cambridge University Press, 2011), 7 edn.

3. Ghosh, N. & Vitkin, I. A. Tissue polarimetry: concepts, challenges, applications, and outlook. *Journal of Biomedical Optics* **16**, 110801 (2011).

4. Beuthan, J., Minet, O., Helfmann, J., Herrig, M. & Müller, G. The spatial variation of the refractive index in biological cells. *Physics in Medicine and Biology* **41**, 369–382 (1996).

5. Menzel, M. *et al.* A Jones matrix formalism for simulating three-dimensional polarized light imaging of brain tissue. *Journal of the Royal Society Interface* **12**, 20150734 (2015).

6. Demtröder, W. *Experimentalphysik 2 – Elektrizität und Optik* (Springer-Verlag Berlin Heidelberg, 2009), 5 edn.
